# “Of Marine Mammal Neuroscience and Men”: Needs and Perspectives in Marine Mammal Neuroscience

**DOI:** 10.1002/cne.70067

**Published:** 2025-07-08

**Authors:** Ksenia Orekhova, Mark Dagleish, Nina Patzke, Simona Sacchini, Federica Giorda, Giovanni Di Guardo, Camilla Testori, Alice Affatati, Tommaso Gerussi, Mari Ochiai, Jean‐Marie Graïc

**Affiliations:** ^1^ Department of Comparative Biomedicine and Food Science University of Padova Padova Italy; ^2^ Division of Veterinary Pathology, Public Health and Disease Investigation, School of Biodiversity, One Health and Veterinary Medicine University of Glasgow Glasgow UK; ^3^ Faculty of Medicine, Institute of Mind, Brain and Behavior Health and Medical University Potsdam Germany; ^4^ Department of Morphology, Faculty of Health Sciences Universidad de Las Palmas de Gran Canaria (ULPGC), Campus Universitario de San Cristóbal Las Palmas de Gran Canaria Spain; ^5^ Istituto Zooprofilattico Sperimentale del Piemonte, Liguria e Valle d'Aosta–WOAH Collaborating Centre for the Health of Marine Mammals Turin Italy; ^6^ Veterinary Medical Faculty of the University of Teramo Teramo Italy; ^7^ National Institute of Oceanography and Applied Geophysics—OGS Trieste Italy; ^8^ Department of Mathematics, Informatics and Geosciences University of Trieste Trieste Italy; ^9^ School of Life and Environmental Science Azabu University Kanagawa Japan

**Keywords:** community, marine mammals, multidisciplinary collaboration, neuroanatomy, neuroimaging, neuropathology, neuroscience

## Abstract

As neuroscience techniques become increasingly sophisticatedand accessible, their application to marine mammal research remainsunderdeveloped and fragmented. Cetacean and pinniped brains exhibit remarkableevolutionary specializations; yet systematic, reproducible data across speciesare scarce. Ethical, logistical, and methodological constraints hinder samplingand analysis of central nervous system tissues, often limiting studies to smallcohorts and reducing diagnostic accuracy in neuropathological investigations.Gaps persist in understanding neuroanatomical organization, pathogeneticmechanisms of neurodegeneration, and the effects of acoustic and environmentalstressors on brain health. Noninvasive neuroimaging methods such as post‐mortemmagnetic resonance imaging and diffusion‐weighted imaging offer promise butsuffer from incompatible protocols and limited standardization. In‐vitro andmolecular techniques including cellular reprogramming may provide new avenuesfor translational research if harmonized approaches are adopted. We identify a criticalneed for coordinated efforts to standardize best practice protocols for the sampling, storage and systematic analyses of marine mammal nervous tissues. To this end, we propose the formation of an inclusive, multidisciplinary network and invitecollaboration through our Open Science Framework project. By aligning methodologies and broadeninginternational partnerships, we aim to transform marine mammal neuroscience intoa robust contributor to comparative neurobiology and environmental healthmonitoring. This is a call to action to collectively grow this emerging field.

## Graphical abstract

With cutting‐edge scientific techniques increasingly available to investigate the central nervous systems of marine mammals, scientists from multiple disciplines are encouraged to collaborate on “Best Practices in Sampling, Storage, and Analyses of Marine Mammal Nervous Tissues” (https://osf.io/e6zg2/?view_only=3ce751a651f245d2a374c6c7dba59e0a) according to FAIR (findable, accessible, interoperable, reusable) principles via the Open Science Framework (https://osf.io/). By increasing collaboration among themselves and conventional neuroscientists, as well as standardizing methodologies, marine mammal specialists should strive to improve the quality and comparability of their neuroscientific work.

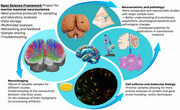

As cutting‐edge techniques in neuroscience become increasingly available to marine scientists (Van Cise et al. 2024), an integrated approach to maximize information from marine mammals’ central nervous system (CNS) samples is required. Significant studies on cetacean macroscopic and microscopic neuroanatomy, neurophysiology, and pathology exist (Lende and Akdikmen 1968; Lende and Welker 1972; Ladygina and Supin 1977; Jacobs et al. 1971, 1979; Bullock and Gurevich, 1979; Glezer et al. 1987, 1993, 1995, 1998; Morgane et al. 1985, 1986; Hof et al. 2005, 1992; Oelschläger 2000; Oelschläger et al. 2008; Poth et al. 2005; Manger et al. 2003; Manger 2006; Kern et al. 2011; Ridgway et al. 2006, 2018, 2019; Mortensen et al. 2014; Parolisi et al. 2018; Raghanti et al. 2019). However, few species, using small sample cohorts within each species, mostly belonging to the Delphinidae family, have been investigated (Figure 1). Very few studies are reproducible due to ethical boundaries (e.g., invasive procedures on live animals), the complicated logistics of obtaining fresh samples from stranded cetaceans, the destructive nature of many analyses, and the impracticality of outdated protocols.

**FIGURE 1:**
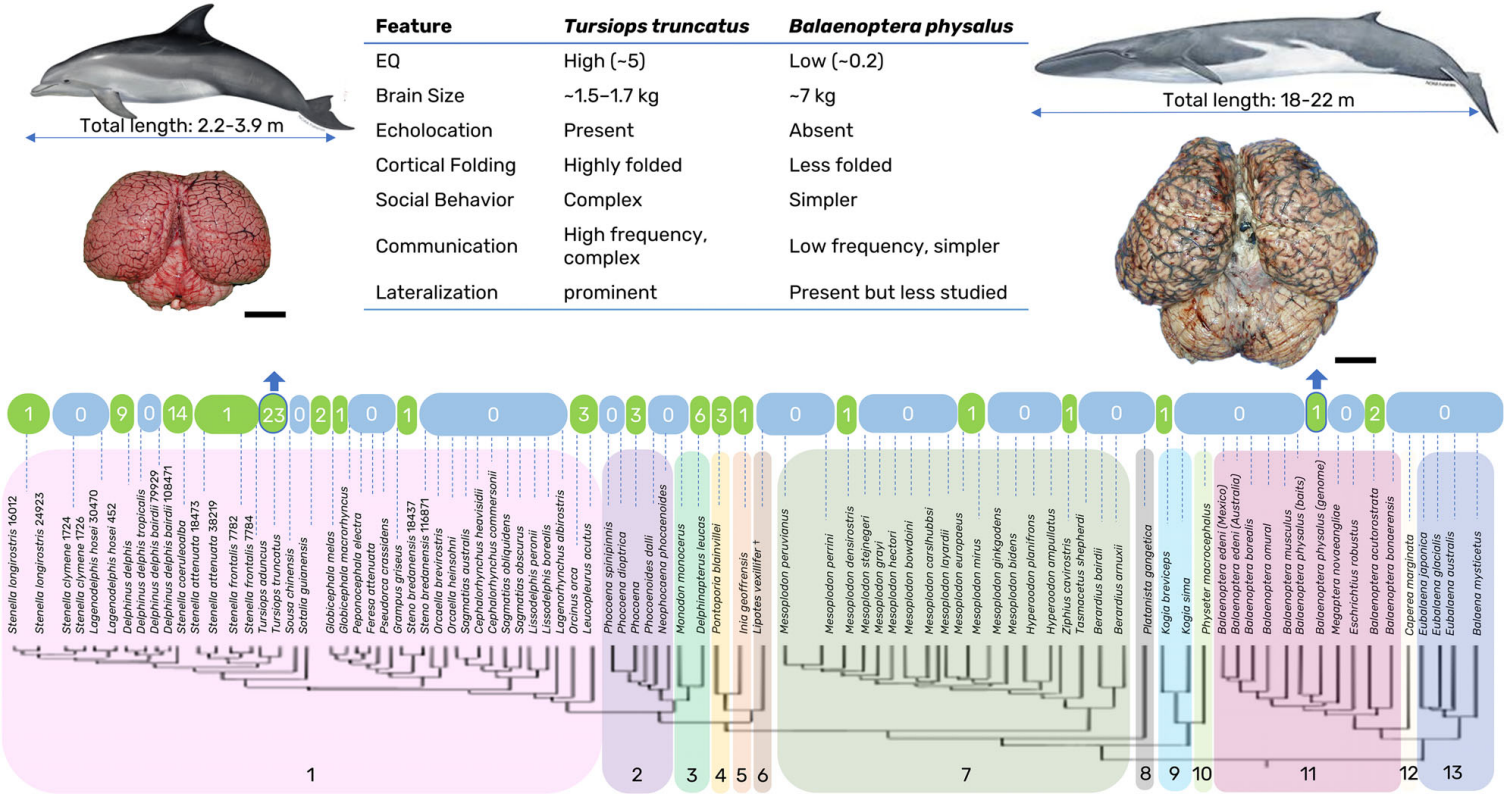
Phylogenomic tree of cetaceans modified from McGowen et al. (2020). The green and blue bubbles indicate the number of studies found on Scopus with the search words: “Species’ Latin name” AND “neuroscience” OR “neuroanatomy” OR “neuro AND imaging” OR “neuropathology.” This is not a comprehensive review, but demonstrates that the majority of neuroscientific studies focus on members of the Delfinidae family (highlighted in pink), leaving large knowledge gaps for many species, particularly mysticetes. A bottlenose dolphin (*Tursiops truncatus*) and fin whale (*Baleaenoptera physalus*) are shown as examples of diverging brain size (drawings are from fisheries.noaa.gov), structure, and functional specialization, reflecting their different ecologies, behaviors, and evolutionary paths. Scale bar: 5 cm. EQ—Encephalization Quotient. Cetacean families are codified by black numbers: 1—Delfinidae; 2—Phocoenidae; 3—Monodontidae; 4—Pontoporiidae; 5—Iniidae; 6—Lipotidae (*Lipotes vexillifer* considered functionally extinct^†^); 7—Ziphiidae; 8—Platanistidae; 9—Kogiidae; 10—Physeteridae; 11—Balaenopteridae and Eschrichtidae; 12—Neobalaenidae; 13—Balaenidae.

Previous book chapters (De Graaf 1967; Pilleri and Gihr 1970; Morgane and Glezer 1990; Ridgway 2000; Cozzi et al. 2017; Huggenberger et al. 2018; Cook et al. 2024) and scientific journal publications (Ridgway 1988; Oelschläger 2008; Morgane et al. 1980; De Vreese et al. 2023) have highlighted deficient areas and specific questions to address in future works (see references for comprehensive review). Here, we summarize the most pressing issues and research questions and propose closer international collaboration to promote the synergy of expertise into the growing field of marine mammal neuroscience. We suggest to do this by sharing best practices and information via the Open Science Framework.

## 1 Main Issues

### 1.1 Neuroanatomy and Neurophysiology

While cetacean brains adhere to the general mammalian blueprint (Jacobs et al. 1979; Oelschläger 2008), striking anatomical specializations imply that cetacean functional brain areas differ from most terrestrial mammals: (1) The auditory pathway in odontocetes is much more developed than in primates, with striking parallels between echolocating odontocetes and bats (Li et al. 2010; Parker et al. 2013; Morell et al. 2020; Ketten et al. 2021; Moss et al. 2023), reflecting the high level of adaptation to predominantly acoustic sensory input (Li et al. 2010); (2) The allocortex is notably small, including a lack/reduction of olfactory structures (Oelschläger 2008; Breathnach 1960) and underdeveloped cetacean hippocampus (Patzke et al. 2015) and limbic lobe; (3) The periallocortex and striatum are very developed compared to any terrestrial mammal, implying their involvement in an alternative pathway for processing emotions, memory, and learning, possibly compensating for the reduced limbic structures (Jacobs et al. 1971, 1979; Morgane et al. 1980; De Vreese et al. 2023); (4) the cerebral cortex is thin and extensively folded with a remarkably large surface area. The cortex contains large, rounded pyramidal neurons, and layer IV is rudimentary/absent, hence thalamic input pathways differ from primates (Jacobs et al. 1979; Hof et al. 1992). Most neuroanatomical studies remain largely qualitative, and while useful given the species’ variable evolutionary adaptations, stereological and morphometric techniques would enable a standardized, reproducible approach to determine physiological baselines for meaningful translational comparisons. More species, notably mysticetes and pinnipeds, should be included in systematic analyses on cytoarchitecture, neurochemistry, and connections of the cortex and brainstem. We should also study how marine mammals regulate their vital functions by investigating microvascularization and neuronal mechanisms mitigating hypoxia during deep dives.

For a handful of pinniped species, initial morphological, neurochemical, molecular, and electrophysiological analyses of primary sensory brain areas exist (Sawyer et al. 2016; Turner et al. 2017; Hanke and Reichmuth 2022; Graïc et al. 2024; Geßner et al. 2022; Cook et al. 2021)^,^ notably, a magnetic resonance imaging (MRI)‐based atlas of the gray seal brain (Hoeksema et al. 2021). Detailed analyses of the pinniped brain should follow, including microscopic descriptions of internal structures. Functional MRI, near‐infrared spectroscopy, and other in vivo techniques promise to glean physio‐anatomical insights into pinniped brain evolution.

### 1.2 Neuropathology

Just like humans, many marine mammals are apex predators constantly exposed to variably polluted environments over long timescales (Di Guardo 2018) making them useful indicators for bioaccumulation, pollution, and disease (Davis et al. 2021; Garamszegi et al. 2024; Sonne et al. 2020; Gonzalvo et al. 2015). Neuroinflammation is a leading cause of death in stranded cetaceans, with the brain sometimes being the only affected organ (Sierra et al. 2020; Giorda et al. 2022). We consider the main issues in marine mammal neuropathology to be: (1) CNS collection is not routinely performed or undertaken erroneously due to logistics or lack of knowledge. Typically, the brain is extracted toward the end of a necropsy compromising tissue preservation, as it is prone to rapid autolysis (Encha‐Razavi 2003). Therefore, CNS disorders are frequently overlooked and underestimated; (2) apart from well‐known cetacean neurotropic pathogens, including cetacean morbillivirus (CeMV), *Brucella ceti*, herpesviruses, and *Toxoplasma gondii* (Vargas‐Castro et al. 2021; Fernández‐Escobar et al. 2022; Grattarola et al. 2023), some zoonotic pathogens, such as highly pathogenic avian influenza A(H5N1) virus (clade 2.3.4.4b) (Thorsson et al. 2023; Murawski et al. 2024), are of concern but not screened for routinely; (3) regarding neurodegenerative diseases (NDDs) and selective neuronal vulnerability in the absence of an infectious etiology, almost only delphinids have been investigated (Davis et al. 2021; Garamszegi et al. 2024). The advantages of transgenic animals are indisputable, but spontaneously occurring terrestrial (Ackermans et al. 2021, 2022; Domínguez‐Oliva et al. 2023; Tan et al. 2024) and marine (Garamszegi et al. 2024; Sacchini et al. 2020; Vacher et al. 2023; Orekhova et al. 2024; Venn‐Watson and Jensen 2025) mammal models may yield more pertinent data on the physiopathology of human NDDs. However, we must be careful not to oversell marine mammals as reliable translational models for human NDDs before more extensive behavioral, biomolecular, and phenotypical characterizations of Alzheimer's disease (AD)‐like and other NDD‐like pathologies are completed; (4) despite marine mammals relying on sound generation and hearing for their survival (Southall et al. 2019), neurodegeneration at morphological and molecular levels has yet to be conclusively correlated to acoustic overexposure (Kujawa and Liberman 2009).

As such, we consider the most pressing neuropathological study topics to be as follows: compiling best practice protocols for representative CNS sampling to achieve a diagnosis and insights into the pathogenesis of the various disease processes affecting marine mammals' CNS; deeper studies into the pathogenesis of infectious neuropathies, such as cellular prion protein as a putative receptor for *B. ceti* (Watarai et al. 2003; Angelucci et al. 2022), or phosphoprotein, matrix, and fusion protein genes linked to CeMV spread (Domingo et al. 1995; Di Guardo et al. 2011; 2016; Di Guardo and Mazzariol 2016; Lucá et al. 2017; Wessels et al. 2021); determining the causes of selective neuronal vulnerability to establish links between human and cetacean CNS disorders (Vacher et al. 2023; Orekhova et al. 2024; Venn‐Watson and Jensen 2025; Watanabe et al. 2019; Zinzula et al. 2022; Di Guardo 2023); developing a standardized behavioral assessment protocol to correlate cognitive performance to concomitant neuropathology, and analyzing the effects of anthropogenic underwater noise based on existing knowledge of marine mammal auditory pathways and integration with environmental and acoustic data (Ridgway 2000; Ogawa and Arifuku 1948; Malkemper et al. 2012; Duarte et al. 2021; Morell et al. 2017; Nieder et al. 2022; Orekhova, Selmanovic, et al. 2022; Orekhova, Centelleghe, et al. 2022). Importantly, to minimize the negative impact of postmortem autolysis when using opportunistic samples from stranded cetaceans, the brain and spinal cord should be collected as soon as possible during the necropsy of marine mammals (not at the end).

We should strive toward establishing the physiological baseline of each species, that is the morphological, neurochemical, and gene expression profiles of representative CNS areas using uniform techniques, and ensure a nuanced discussion of findings that seem to deviate from an, as yet, insufficiently studied norm. Lesion sites must be anatomically referenced. Systematically validating a selection of biomarkers of protein misfolding and aggregation, neuroinflammation, apoptosis, autophagy, and CNS remodeling would establish reliable baselines and facilitate ongoing monitoring of impacts, including climate change, on marine mammals (Orekhova, Centelleghe, et al. 2022; Bongioanni et al. 2022).

### 1.3 Neuroimaging

Compared to pinnipeds, conducting advanced, invasive, in vivo techniques on cetacean brains has significant practical and ethical challenges. Therefore, noninvasive methods, for example, postmortem MRI including diffusion‐weighted imaging (DWI), are invaluable. MRI and DWI capture high resolution whole‐brain images in situ, allowing examination of intact morphology, intracerebral white matter connections, and total brain and brain region volume for assessment of neuromorphology (Orekhova, Selmanovic, et al. 2022; Marino et al. 2001; Montie et al. 2008; Oelschläger et al. 2010; Berns et al. 2015; Wright et al. 2018; Gerussi et al. 2023). They help to identify and understand various pathologies (Cook et al. 2018, 2021; Montie et al. 2009). The main neuroimaging‐related issues are: (1) low applicability of live‐animal imaging due to ethical and practical constraints; (2) variable feasibility of postmortem MRI scans with a minimum power of 3 Tesla (T), which is required to achieve basic image quality. MRI scanners of 3 T are the most widespread and are big enough to accommodate whole heads of small odontocetes and pinnipeds or extracted brains of larger cetaceans. However, scanning a brain/head depends on geographic region, logistics, and carcass decomposition, with consequent (3) incompatibility of imaging requirements with conventional diagnostic sampling of the CNS; (4) lack of standardized MRI/DWI protocols. For these reasons, no detailed cetacean brain atlas exists. We suggest a discussion between scientists regarding best practices that may reconcile the need to maintain the brain/head whole to avoid alterations in morphology and disruption of neural pathways for scanning and to adequately sample for other postmortem analyses.

### 1.4 In Vitro and Molecular Techniques

A key challenge in marine mammal neuroscience is the difficulty of establishing primary cultures of CNS‐derived cells, such as neurons and glial cells, which limits our ability to study dynamic cellular processes. While fibroblasts and peripheral blood mononuclear cells from marine mammals have been successfully cultivated for cytotoxicity and immunotoxicity studies (Bjørneset et al. 2023; Ochiai et al. 2020; Desforges et al. 2016), CNS cells remain elusive.

Recent advancements in cellular reprogramming, including induced pluripotent stem cells (iPS cells) (Takahashi and Yamanaka 2006; Yu et al. 2007) and direct reprogramming (Wang et al. 2021), offer promising pathways for generating neural cells from marine mammals (Ochiai et al. 2021) to overcome previous limitations. Future research should focus on refining these techniques, enhancing their reproducibility and scalability, and applying them to neurotoxicology and marine mammal CNS studies.

Additionally, preserving marine mammal brain tissues in RNALater or cryoprotective solutions enables long‐term molecular analyses. Standardizing tissue preservation protocols will be crucial for ensuring comparability across studies; advancing research into neurodegeneration, neurotoxicity, and environmental stress responses; and providing refined models for broader CNS studies across species.

## 2 Call for Action

While the neuroscientific subfields mentioned above identify specific questions, the issues they all share are the need for a more systematic, reproducible, and transparent approach using larger sample sizes, multidisciplinary expertise, and compatible, validated protocols.

We therefore call for the following main actions:

1. to create best‐practice guidelines for the standardized sampling, storage and multidisciplinary investigations of marine mammal nervous tissues (within a year from the publication of this commentary) and
2. to increase the number of institutions involved and the collaborations between them (not to create only one hub).

To fulfil these actions, we have made our Open Science Framework project public (“Best Practices in Sampling, Storage, and Analyses of Marine Mammal Nervous Tissues”: and invite marine mammal and neuroscience experts to collaborate with us via this platform based on FAIR principles.

The aim is not to become the big neuroscience fish in the small marine mammal pond, but to become the small marine mammal neuroscience fish in the big neuroscience ocean. Establishing an inclusive, multidisciplinary international community of marine mammal neuroscientists adapting state‐of‐the‐art techniques, we will synergistically advance and maximize knowledge in this nascent field.

## Author Contributions

All authors contributed equally to the writing and editing of this manuscript, and are listed in order of text/edit appearance. Ksenia Orekhova, Jean‐Marie Graïc, and Giovanni Di Guardo organized the workshop “Neuroscience as an emerging field in marine mammalogy” at the 2024 European Cetacean Society Conference and subsequent working group correspondence that led to the creation of this commentary.

## Data Availability

The authors have nothing to report.
